# Bile Acids and Risk of Adverse Cardiovascular Events and All-Cause Mortality in Patients with Acute Coronary Syndrome

**DOI:** 10.3390/nu16071062

**Published:** 2024-04-05

**Authors:** Javier Mateu-Fabregat, Hamza Mostafa, Raul Sanchez-Gimenez, Óscar M. Peiró, Gil Bonet, Anna Carrasquer, Georgios A. Fragkiadakis, Alfredo Bardaji, Mònica Bulló, Christopher Papandreou

**Affiliations:** 1 Nutrition and Metabolic Health Research Group, Department of Biochemistry and Biotechnology, Rovira i Virgili University (URV), 43201 Reus, Spain; javier.mateu@urv.cat (J.M.-F.); monica.bullo@urv.cat (M.B.); 2 Institute of Health Pere Virgili (IISPV), 43204 Reus, Spain; raul.sagi@hotmail.com (R.S.-G.); opi220290@gmail.com (Ó.M.P.); gil.bonet.p@gmail.com (G.B.); carrasquer1987@gmail.com (A.C.); abardaji.hj23.ics@gencat.cat (A.B.); 3 Center of Environmental, Food and Toxicological Technology—TecnATox, Rovira i Virgili University, 43201 Reus, Spain; 4 Department of Cardiology, Joan XXIII University Hospital, 43005 Tarragona, Spain; 5 Department of Medicine and Surgery, Rovira i Virgili University, 43005 Tarragona, Spain; 6 Department of Nutrition and Dietetics Sciences, School of Health Sciences, Hellenic Mediterranean University, 72300 Siteia, Greece; fragkiadakis@hmu.gr; 7 CIBER Physiology of Obesity and Nutrition (CIBEROBN), Carlos III Health Institute, 28029 Madrid, Spain

**Keywords:** primary bile acids, secondary bile acids, acute coronary syndrome, major adverse cardiovascular events

## Abstract

The relationship between bile acids (BAs) and adverse cardiovascular events following acute coronary syndrome (ACS) have been little investigated. We aimed to examine the associations of BAs with the risk of cardiovascular events and all-cause mortality in ACS. We conducted a prospective study on 309 ACS patients who were followed for 10 years. Plasma BAs were quantified by liquid chromatography coupled to tandem mass spectrometry. Cox regression analyses with elastic net penalties were performed to associate BAs with MACE and all-cause mortality. Weighted scores were computed using the 100 iterated coefficients corresponding to each selected BA, and the associations of these scores with these adverse outcomes were assessed using multivariable Cox regression models. A panel of 10 BAs was significantly associated with the increased risk of MACE. The hazard ratio of MACE per SD increase in the estimated BA score was 1.35 (95% CI 1.12–1.63). Furthermore, four BAs were selected from the elastic net model for all-cause mortality, although their weighted score was not independently associated with mortality. Our findings indicate that primary and secondary BAs may play a significant role in the development of MACE. This insight holds potential for developing strategies to manage ACS and prevent adverse outcomes.

## 1. Introduction

Acute Coronary Syndrome (ACS) is a critical cardiovascular condition that demands immediate attention and comprehensive management [1,2]. ACS encompasses a spectrum of cardiac ischemic events, including unstable angina, non-ST-segment elevation myocardial infarction (NSTEMI), and ST-segment elevation myocardial infarction (STEMI) [1,3], and is associated with the increased risk of cardiovascular events and mortality. To improve management and therapy of ACS, effective tools for risk assessment in secondary prevention are needed. Since ACS frequently exhibits metabolic alterations, a metabolomics approach might play a significant role in the better management of ACS patients, by advancing our understanding of the mechanisms underlying the increased risk of recurrent cardiovascular events following ACS and helping in the identification of biomarkers related to these adverse events [4].

Few studies have investigated the relationship between circulating metabolites and adverse events after ACS. For instance, in patients with ACS, serum *N*-acetylneuraminic has been related to myocardial injury and the degree of coronary lesions, resulting in the possibility to use it as a biomarker for ACS patient risk assessment [5]. Other studies have reported associations of plasma tricarboxylic acid (TCA) cycle metabolites and plasma trimethylamine-*N*-oxide (TMAO) and its precursors with the risk of cardiovascular events in ACS patients [3,6]. Recently, gut microbiota-derived metabolites have attracted considerable attention for cardiovascular health [7,8]. An example of these metabolites is the bile acids (BAs). BAs are divided into primary (PBAs) and secondary bile acids (SBAs). PBAs are the end-product of cholesterol metabolism and are synthesized in the liver, whereas SBAs are produced in the colon by the gut microbiota metabolism, from PBAs as substrates [9,10]. In chronic heart failure (CHF) patients, an elevation in gut microbiota-derived SBAs has been observed [11]. On the other hand, in ACS patients, particularly in acute myocardial infarction (AMI), a lower level of serum total BAs (TBA) was significantly linked to cardiovascular diseases (CVD) and all-cause mortality [12]. However, in the latter study, the particular BA components which may offer further insights into understanding the pathophysiological processes relevant to recurrent events in patients presenting with ACS were not examined. Additionally, results from recent cohort studies regarding SBAs in relation to CVD, as well as CVD/all-cause mortality, were inconsistent [7].

To date, there is scarce evidence about the associations between BAs and the risk of recurrent cardiovascular events and mortality in ACS patients. We hypothesized that circulating PBAs, along with their gut microbiota-derived SBAs are associated with adverse outcomes following ACS. Therefore, the aim of this study was to investigate the association of plasma PBAs and their gut microbiota-derived SBAs with subsequent major adverse cardiovascular events (MACE) and all-cause mortality in patients with ACS.

## 2. Materials and Methods

### 2.1. Study Design and Population

The study was conducted among patients with ACS who attended the Joan XXIII University Hospital in Tarragona, Spain, between January 2011 and May 2013. Included participants underwent a coronary angiography and were followed up until 2022. ACS was defined as patients with ST-segment elevation myocardial infarction (STEMI), non-ST segment elevation myocardial infarction (NSTEMI) and unstable angina, in accordance with the guidelines set forth by the European Society of Cardiology. The current universal definition of myocardial infarction was used to diagnose acute myocardial infarction (STEMI or NSTEMI) [13]. Patients who suffered a non-type 1 myocardial infarction were further excluded. A detailed explanation of the definition of ACS can be found elsewhere [3,6]. After excluding those patients who did not have blood samples (*n* = 31), a total of 309 participants remained in the analysis (Supplementary Materials Figure S1) [14]. The Institutional Ethical Committee approved the study protocol and all participants provided informed written consent. This study was conducted in accordance with the Declaration of Helsinki.

### 2.2. Ascertainment of the Outcomes

All data regarding the clinical outcomes were obtained by yearly contact with the patients and analyzing the hospital’s patient management information system [myocardial infarction (ICD-10-CM I21), and hospitalization for heart failure (ICD-10-CM I50)]. MACE was defined as the composite of myocardial infarction, hospitalization for heart failure, and all-cause mortality. All-cause mortality consisted of cardiac, vascular (including stroke, acute pulmonary oedema, non-ischemic fatal arrythmia) and non-cardiovascular causes.

### 2.3. Bile Acids Profiling

Plasma aliquots derived from blood samples collected from the patients during the coronary angiography in a fasting state were stored at −80 °C until analysis at the Biobank of the Pere Virgili Health Research Institute. Bile acids were quantified by the stable-isotope dilution method using liquid chromatography coupled to tandem mass spectrometry. For sample preparation, 100 μL plasma were aliquoted into a 1.5 mL Eppendorf tube and mixed with 400 μL of ACN containing internal standards (0.2 μM CA-d5, 0.1 μM conjugated mix). Samples were vortexed and centrifuged for 10 min at 15,000× rpm and 4 °C. Supernatants were transferred to a new tube and were evaporated in a SpeedVac at 45 °C. Samples were reconstituted with 50 μL of methanol:water (1:1, *v*/*v*) and transferred to glass vials for their analysis. To separate the several compounds, samples were chromatographed on a Kinetex EVO C18 (150 × 2.1 mm) from Phenomenex (Torrance, CA, USA). The gradient consisted of 25% B for 0.08 min, to 30% B at 7.2 min, to 50% B at 13.20 min, to 100% B at 13.50 min, kept at 100% B for 1.5 min, to 25% B at 15.50 min for 2 min. Mobile phase A was 0.1% ammonium hydroxide and 10 mM ammonium acetate in water, and B was acetonitrile. The flow rate was kept constant at 0.4 mL/min, sample injection volume was 2 μL, and the column temperature was set at 27 °C for the duration of the sequence.

Agilent QqQ/MS 6490 Series with an electrospray ionization probe operating in negative ion mode was used for mass spectrometric analysis. The source conditions were set at 20 psi for the nebulizer gas, 200 °C for the gas temperature, 14 L/min for the gas flow, 250 °C for the sheath gas temperature, 11 L/min for the sheath gas flow, 3000 V for the capillary voltage, and 1500 V for the nozzle voltage. Quantitative determination was performed using the multiple reaction monitoring mode, and the transitions for each compound are detailed in Supplementary Materials (Table S1). Assay quality assurance was monitored by routine analysis of pooled quality control plasma. Information about the mass to charge ratio and retention time is shown in Supplementary Materials Table S1 [13].

### 2.4. Covariate Assessment

Information on demographics, smoking status, medication, type 2 diabetes (T2D), dyslipidemia, and hypertension were recorded during hospital admission. Subjects were considered to have type 2 diabetes, dyslipidemia, and hypertension if they had previously been diagnosed or they consumed antidiabetic, cholesterol-lowering, and antihypertensive agents, respectively. Body mass index (BMI) was calculated by dividing weight by height squared (kg/m^2^). To calculate the estimated glomerular filtration rate (eGFR), the Chronic Kidney Disease Epidemiology Collaboration creatinine equation was employed [15].

### 2.5. Statistical Analyses

Clinical and sociodemographic data of study population are detailed as mean ± standard deviation or median (interquartile range) for continuous data and percentages for categorical variables. One out of the 16 bile acids, namely tauroursodeoxycholic acid, was removed because of the number of missing values (>20%). Remaining metabolites presented an average percentage of missingness (min, max) equal to 1.87 (0.00, 8.73) (Supplementary Materials Figure S2) [14]. As recommended for metabolomic studies [16], we applied the random forest imputation approach (“missForest” R package (v.1.5)) to deal with the missing values on the remaining BAs. The inverse normal transformation, which generates a rank-based standard normal distribution (mean = 0, SD = 1), was applied to the 15 BAs. Regarding covariates, missing data in BMI was imputed using the multivariate imputation chained equations package (“mice” R package (v. 3.15.0)) (Supplementary Materials Figure S3) [14]. Density plots of observed and imputed data of all the BAs and BMI were carried out to check the effectiveness of the imputation as shown in Supplementary Materials Figure S4 [14].

#### 2.5.1. Univariate Analyses

Unadjusted and multivariable Cox proportional hazard models were used to examine the individual associations (per 1 SD increment) of BAs with the risk of MACE and all-cause mortality. Hazard ratios (HR) and their 95% confidence intervals (CI) were estimated. The multivariable model was adjusted for several potential confounders based on previous research: age, sex, BMI, smoking, hypertension, dyslipidemia, T2D, unstable angina, STEMI, non-STEMI, statin medication, beta-blockers, oral antidiabetic medication, insulin medication, diuretics, aspirin and eGFR. The Benjamini–Hochberg false discovery rate procedure was used to account for multiple testing and its statistical significance was set at *p*-value < 0.05.

#### 2.5.2. Multivariate Analyses

We randomly split 80% of the data into the training set and the remaining 20% sample into the testing set to develop and test BA related prediction models for MACE and all-cause mortality. Considering the high dimensionality and collinear nature of the data (Supplementary Materials Figure S5) [14], we performed Cox regression analyses with elastic net penalties (“glmnet” package (v. 4.1-7)). Elastic net combines the L1 (lasso) and the L2 (ridge) penalties, to select for groups of correlated variables while shrinking the coefficients of redundant variables to zero, thereby retaining only the important ones [17]. Then, we applied the model with 10-fold cross-validation (CV) on the training set and estimated the optimal value for alpha (α), from 0.1 to 1 in 0.05 increments, as well as for the tuning parameter [λ (lambda)]. We also computed the concordance index (*C*-index) on the testing set. We selected the optimal values of alpha and lambda based on the combination that yielded a higher *C*-index in the test set. We applied the selected alpha (for MACE: 0.1, for all-cause mortality: 0.15) and lambda (for MACE: 0.002765204, for all-cause mortality: 0.011728859) values to each elastic net regression for every training set in a 100-iteration loop. We built the BAs model only with those BAs that were consistently selected in 100 iterations. We calculated the mean and 95% CI of the regression coefficients of the selected BAs from the elastic net model and then we created a BA score as the weighted sum of the average elastic net regression coefficients from the 100 iterations. The BA score was further standardized with its z-score (mean = 0; SD = 1) and we ran multivariable Cox regression analyses to explore the independent associations of the identified BA score (per 1 SD increment) with MACE and all-cause mortality. Multivariable models were adjusted for the same potential confounders previously mentioned in the univariate analyses. HR and their 95% CI were calculated. All statistical analyses were carried out using R statistical package (v.4.2.2) (R Foundation for Statistical Computing, Vienna, Austria) and a *p*-value < 0.05 was considered as statistically significant.

## 3. Results

### 3.1. Baseline Characteristics of the Population

The general characteristics of the study population are described in Table 1. The mean age was 64.9 ± 12.3 years, and the majority of the population were men (71.2%). Participants were mostly overweight and one-third were current smokers. While some had type 2 diabetes (37.2%), most of them had hypertension (67.6%) and dyslipidemia (60.8%).

About one-third of the population are current smokers. Altogether, 62% of patients were diagnosed with NSTEMI, while 22% and 15% were admitted with STEMI and unstable angina, respectively. Likewise, a significant proportion of the population was taking medication such as statins, beta-blockers, and aspirin among others. The median (interquartile range) of the BA concentrations (including PBAs and SBAs) can be found in the Supplementary Materials Table S2 [14].

### 3.2. Association of Bile Acids with MACE

**Figure 1:**
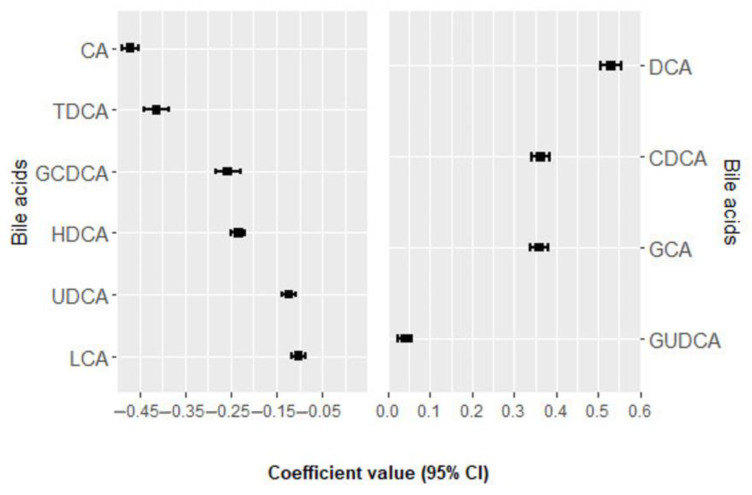
Bile acids ranked from the highest to the lowest elastic net positive and negative regression coefficients for MACE. Bile acids with negative regression coefficients (m = 6) are plotted in the left part, whereas those with positive regression coefficients (m = 4) are shown in the right part. Exposure contrast is per SD/z-score increase in the bile acid. Abbreviations: CA, cholic acid; CDCA, chenodeoxycholic acid; DCA, deoxycholic acid; GCA, glycocholic acid; GCDCA, glycochenodeoxycholic acid; GUDCA, glycoursodeoxycholic acid; HDCA, hyodeoxycholic acid; LCA, lithocholic acid; MACE, major adverse cardiovascular events; TDCA, taurodeoxycholic acid; UDCA, ursodeoxycholic acid.

Within a mean follow-up period of 6.7± 3.6 years, a total of 131 incident MACE events occurred. In the univariate analysis, none of the bile acids were significantly associated with MACE after adjustment for multiple comparisons (Supplementary Materials Table S3) [14]. In multivariate analysis, 10 bile acids were selected 100 times from the elastic net regression and their positive and negative regression coefficients for MACE, as shown in Figure 1 and Supplementary Materials Table S4 [14]. Deoxycholic acid (DCA), chenodeoxycholic acid (CDCA), glycocholic acid (GCA), and glycoursodeoxycholic acid (GUDCA) were positively associated with the risk of MACE, while cholic acid (CA), taurodeoxycholic acid (TDCA), glycochenodeoxycholic acid (GCDCA), hyodeoxycholic acid (HDCA), ursodeoxycholic acid (UDCA), and lithocholic acid (LCA) were negatively associated with the risk of MACE. The BA score was consistently associated with an increased risk of MACE in the crude model (HR per 1 SD = 1.47; 95% CI: 1.24, 1.74; *p*-value < 0.001) and in the multivariable model (HR per 1 SD = 1.35; 95% CI: 1.12, 1.63; *p*-value = 0.001) (Table 2).

### 3.3. Association of Bile Acids with All-Cause Mortality

**Figure 2:**
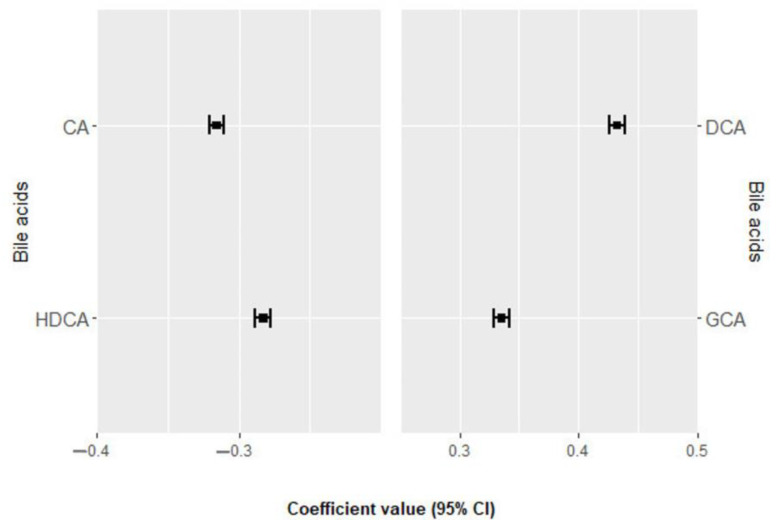
Bile acids ranked from the highest to the lowest elastic net positive and negative regression coefficients for all-cause mortality. Bile acids with negative regression coefficients (m = 2) are plotted in the left part, whereas those with positive regression coefficients (m = 2) are shown in the right part. Exposure contrast is per SD/z-score increase in the bile acid. Abbreviations: CA, cholic acid; DCA, deoxycholic acid; GCA, glycocholic acid; HDCA, hydrodeoxycholic acid.

During an average follow-up of 7.8 ± 3.1 years a total of 90 deaths were recorded, including 35 from cardiovascular causes. None of the bile acids were significantly associated with all-cause mortality in the univariate analysis after Benjamini–Hochberg adjustment (Supplementary Materials Table S5) [13]. Figure 2 and Supplementary Materials Table S6 [13] show the four bile acids selected 100 times in the elastic net regression and their positive and negative regression coefficients for all-cause mortality. DCA and GCA were directly associated with all-cause mortality, while CA and HDCA were negatively associated. The derived BA score was significantly associated with all-cause mortality in the unadjusted model but not in the multivariable model (Table 2).

## 4. Discussion

To the best of our knowledge, this is the first study to explore the associations of plasma PBAs and their gut microbiota-derived SBAs with subsequent MACE and all-cause mortality in patients with ACS. The findings of this prospective cohort study of 309 patients with ACS showed that a profile of 15 PBAs and SBAs was independently associated with the risk of MACE. The unadjusted model displayed a positive association between a BA profile of four BAs and the risk of all-cause mortality. However, after adjusting for potential confounders, the association did not remain significant. This might be explained by the fact that all-cause mortality included non-cardiovascular causes as well. The robustness and the stability of the elastic net Cox regression analysis allowed us to develop a metabolic signature that implicates BA metabolism in the risk of MACE in ACS patients.

To date, evidence on the relationship between BAs and the risk of MACE and mortality in patients with ACS is scarce. One previous study found negative associations between serum TBA levels and the severity of coronary lesions, myocardial damage, and inflammation in ACS [12]. This study also found a protective impact of gut *Lactobacillus* in ACS patients, partially mediated by TBA levels. Gut dysbiosis, associated with various non-communicable diseases, including CVD, cancer, type 2 diabetes, and obesity was observed in ACS patients [18]. These patients had increased intestinal permeability and changes in gut microbiota composition [18]. Similar shifts in gut microbiota composition were noted in atherosclerosis processes [19,20,21,22]. Gut microbiota plays an important role in BA metabolism by deconjugating the PBAs through microbial bile salt hydrolase enzyme, followed by a multi-step process known as 7-α-dehydroxylation, which is mediated by the *Clostridium* subcluster XIVa (XIVa) [9,23,24,25]. According to recent studies on gut dysbiosis, the DCA/(DCA + CA) ratio is thought to be a marker for the 7-α-dehydroxylation process, and a decrease in XIVa is strongly correlated with reduced intestinal 7α-dehydroxylation of PBAs [25,26]. In this present study, DCA had a positive association with MACE while CA had a negative association. The hypothesis proposing the use of PBAs and SBAs ratios as markers for dysbiosis could suggest a potential association between gut dysbiosis and the development of MACE in ACS patients in our study. However, it is imperative to underscore the necessity of a thorough taxonomy analysis to support this hypothesis.

In the present study, two bile acids, one secondary (deoxycholic acid, DCA) and one primary (glycocholic acid, GCA) were positively associated with MACE and all-cause mortality, while two more, one secondary (hyodeoxycholic acid, HDCA) and one primary (cholic acid, CA) were negatively associated. CA is typically converted to DCA and is mainly re-absorbed in the colon and enters enterohepatic circulation, reaching the liver where it is re-conjugated and secreted in bile; while this is an endogenous process, it is highly dependent on and increases with high fat diets, which have well-known negative health effects [27]. Concerning DCA, levels above the median were previously independently associated with higher risks of end-stage kidney disease and all-cause mortality but not with cardiovascular events [28].

Glycocholic acid (GCA) levels in the serum of patients with benign biliary disease, as well as cholangiocarcinoma, were found to be high compared with healthy controls [29]. Furthermore, the serum GCA-to-TBA ratio was independently associated with non-alcoholic fatty liver disease (NAFLD). A simple novel model incorporating the GCA-to-TBA ratio score had a good performance in discriminating NAFLD from the general population [30]. A hypothesis can be made based on the above, that GCA may increase the risk of mortality, through the liver function.

An untargeted metabolomics study conducted on rats to evaluate the synergistic effect of some flavonoids on ticagrelor, an antiplatelet oral therapy that is considered to be one of the first-line oral therapy for atherosclerosis and ACS, found that after enhancing the bioavailability of ticagrelor, the plasma levels of GCA and GUDCA decreased, while DCA levels increased [31]. Another metabolomics study conducted in patients with unstable angina (UA) revealed that GCA could be consider as a plasma metabolic biomarker for this condition [32]. Interestingly, these BAs from these two metabolomics studies were found positively associated with MACE in our study.

On the other hand, previous research has demonstrated that Bas, such as UDCA and TDCA, may have protective effects on heart disease and obesity-related insulin resistance and inflammation, respectively [33,34]. Our results are in line with these findings as these BAs were negatively associated with the development of MACE in ACS patients.

The study’s main strengths are its prospective design, involving a long-term follow-up without dropouts. Additionally, the comprehensive profile of BAs included in our study broadens our understanding of the associations of BAs and subsequent severe recurrent cardiovascular events in patients with ACS. Moreover, the use of the elastic net model performed successfully in the variable selection and model construction increased the robustness of our findings. Considering the limitations, the few numbers of death cases may have limited the statistical power to detect the association between the BA profile and all-cause mortality even though we have tried to address this issue using regularized Cox regression. Another limitation is that we used an elastic net regression that considered linear combinations of BAs, while there might be no linear relations between them. Furthermore, even though we adjusted for several confounders, unmeasured confounding factors may have impacted the results. Finally, although our results were internally validated, the generalizability might be limited, and our findings need to be replicated in other populations.

## 5. Conclusions

This prospective cohort study identified a plasma BA profile of PBAs and SBAs which were found to be associated with the risk of developing MACE in ACS patients. Understanding the significant role of gut microbiota derived metabolites in the development of MACE might be helpful in the establishment of novel approaches to the management of ACS. However, larger studies are needed to confirm these findings.

## Figures and Tables

**Table 1:** Baseline characteristics of the population.

Characteristics	Patients (*n* = 309)
Age, years	64.9 ± 12.3
Men, %	71.2
Body mass index, kg/m^2^	28.1 ± 4.0
eGFR, (mL/min/1.73 m^2^)	81.3 (62.1–96.7)
Type 2 diabetes, *n* (%)	115 (37.2)
Hypertension, *n* (%)	209 (67.6)
Dyslipidemia, *n* (%)	188 (60.8)
Smoking	
Never, *n* (%)	110 (35.6)
Former, *n* (%)	105 (33.9)
Current, *n* (%)	94 (30.4)
Discharge diagnostic	
Unstable angina, *n* (%)	49 (15.9)
STEMI, *n* (%)	68 (22.0)
NSTEMI, *n* (%)	191 (62.0)
Medications	
Statins, *n* (%)	156 (50.5)
Beta-blockers, *n* (%)	94 (30.4)
Aspirin, *n* (%)	125 (40.5)
Diuretics, *n* (%)	78 (25.2)
Oral antidiabetic agents, *n* (%)	70 (22.7)
Insulin medication, *n* (%)	27 (8.7)

Continuous data are described as mean ± standard deviation or median (interquartile range), and categorical variables are presented as %. Abbreviations: eGFR, estimated glomerular filtration rate; STEMI, ST elevation myocardial infarction; NSTEMI, non-ST elevation myocardial infarction.

**Table 2:** Associations of the bile acid score with MACE risk and all-cause mortality. Exposure contrast is per SD/z-score increase in the bile acids score.

Bile Acids Score	HR per 1 SD Increment (95% CI)	*p* Value
MACE		
Crude model	1.47 (1.24, 1.74)	**<0.001**
MV	1.35 (1.12, 1.63)	**0.0014**
All-cause mortality		
Crude model	1.31 (1.059, 1.613)	**0.012**
MV	1.12 (0.87, 1.41)	0.368

MV models were adjusted for the following potential confounders: age, sex, body mass index (kg/m^2^), smoking, hypertension, dyslipidemia, type 2 diabetes, unstable angina, acute ST-segment elevation myocardial infarction, non-ST-segment elevation acute myocardial infarction, statin medication, beta-blockers, oral antidiabetic medication, insulin medication, diuretics, aspirin, and glomerular filtration rate. Abbreviations: CI, confidence interval; HR, hazard ratio; MV, multivariable. Statistically significant *p*-values are shown in bold.

## Data Availability

The data presented in this study are available on request from the corresponding author due to privacy.

## Supplementary Materials

The following supporting information can be downloaded at: https://www.mdpi.com/article/10.3390/nu16071062/s1, Figure S1: Flowchart of study participants; Figure S2: Plot of the 16 bile acids according to the percentage of missingness in 309 patients with ACS; Figure S3: Plot of the covariates according to the percentage of missingness in 309 patients with ACS; Figure S4: Density plots comparing observed and imputed data; Figure S5: Correlation matrix for the 15 bile acids considered in the analysis; Table S1: List of metabolites identified in the LC–MS/MS method; Table S2: Baseline concentrations of the bile acids measured in ACS patients; Table S3: Cox regression analysis examining the individual associations between bile acids and risk of MACE; Table S4: Bile acids ranked from the highest to the lowest elastic net positive and negative regression coefficients for MACE; Table S5: Cox regression analysis examining the individual associations between bile acids and all-cause mortality; Table S6: Bile acids ranked from the highest to the lowest elastic net positive and negative regression coefficients for all-cause mortality.
