# Supplementary material for: Bile Acids and Risk of Adverse Cardiovascular Events and All-Cause Mortality in Patients with Acute Coronary Syndrome
Source: Nutrients. 2024 Apr 5;16(7):1062. doi: 10.3390/nu16071062 (PMC11013079; doi:10.3390/nu16071062)
Supplement: Supplementary file 1 [file nutrients-16-01062-s001.zip › nutrients-2902946-supplementary.pdf]

Supplementary file

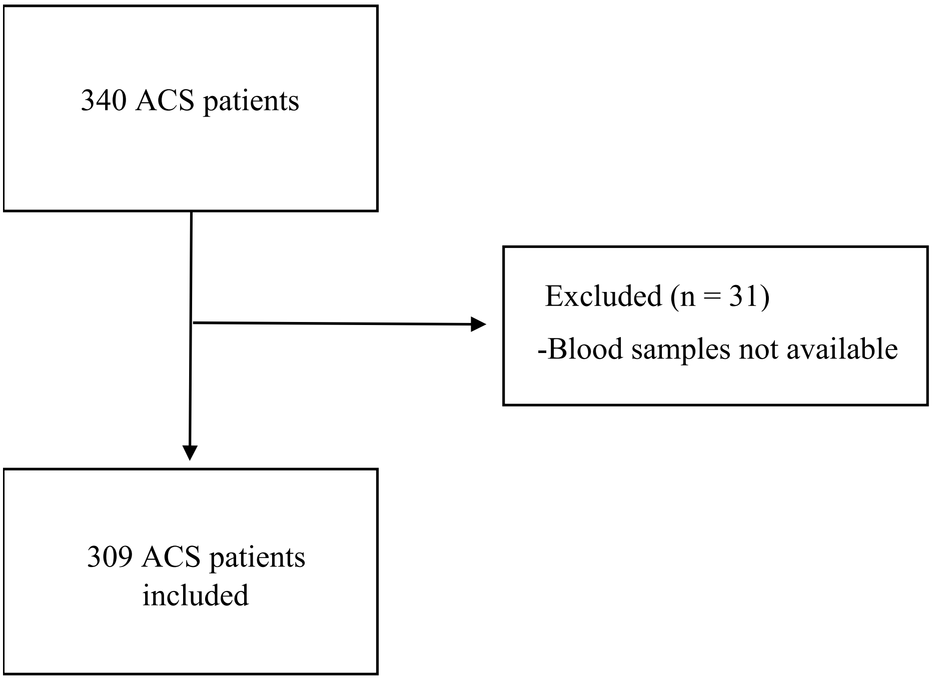

Supplementary Figure S1. Flowchart of study participants.

ACS, acute coronary syndrome.

Supplementary Table S1. List of metabolites identified in the LC-MS/MS method.

Bile acid	HMDB_ID	Retention time (min)	Precursor ion (m/z)	Product ion (m/z)
CA	HMDB0000619	2.001	407.3	343
			407.3	345.3
CA d5	-	1.982	412.3	348
			412.3	350.3
CDCA	HMDB00518	3.928	451.3	391.3
			451.3	373.2
DCA	HMDB00626	4.561	391.3	345.1
			391.3	343.2
GCA	HMDB0000138	2.386	464.3	74
			464.3	402.3
GCA d4	-	2.376	468.3	74
			468.3	406.3
GCDCA	HMDB0000637	4.74	448.3	74
			448.3	386.3
GCDCA d4	-	4.719	452.3	74
			452.3	390.3
GDCA	HMDB0242414	5.375	448.3	74
			448.3	402.3
GDCA d4	-	5.345	452.3	74
			452.3	406.3
GUDCA	HMDB0000708	2.076	448.3	74
			448.3	386.3
			448.3	448.3
GLCA	HMDB0000698	9.208	432.3	74
			432.3	388.3
GLCA d4	-	9.197	436.3	74
			436.3	392.3
GUDCA d4	-	2.064	452.3	74
			452.3	390.3
HDCA	-	2.169	451.3	391.3
			451.3	59
LCA	HMDB00761	8.547	435.3	375.2
			435.3	58.8
TCA	HMDB00036	2.762	514.3	124
			514.3	79.9
TCA d4	-	2.78	518.3	79.9
			518.3	124
TCDCA	HMDB00951	5.576	498.3	79.9
			498.3	124.1

TCDCA d4	-	5.556	502.3	79.9
			502.3	124.1
TDCA	HMDB00896	6.21	498.3	79.9
			498.3	124.1
TDCA d4	-	6.173	502.3	79.9
			502.3	124.1
TLCA	HMDB0000722	9.801	482.3	80
			482.3	124
TLCA d4	-	9.773	486.3	80
			486.3	124
TUDCA d4	-	2.507	502.3	80
			502.3	124.1
TUDCA	HMDB00874	2.526	498.3	124.1
			498.3	80
			498.3	498.3
UDCA	HMDB00946	1.752	451.3	391.3
			451.3	59

Abbreviations: CA, cholic acid; CI, confidence interval; CDCA, chenodeoxycholic acid; TCA, taurocholic acid; DCA, deoxycholic acid; GCDCA, glycochenodeoxycholic acid; GCA, glycocholic acid; GDCA, glycodeoxycholic acid; GLCA, glycolithocholic acid; GUDCA, glycoursodeoxycholic acid; HDCA, hyodeoxycholic acid; LCA, lithocholic acid; TCDCA, taurochenodeoxycholic acid; TDCA, taurodeoxycholic acid; TLCA, taurolithocholic acid; TUDCA, tauroursodeoxycholic acid; UDCA, ursodeoxycholic acid.

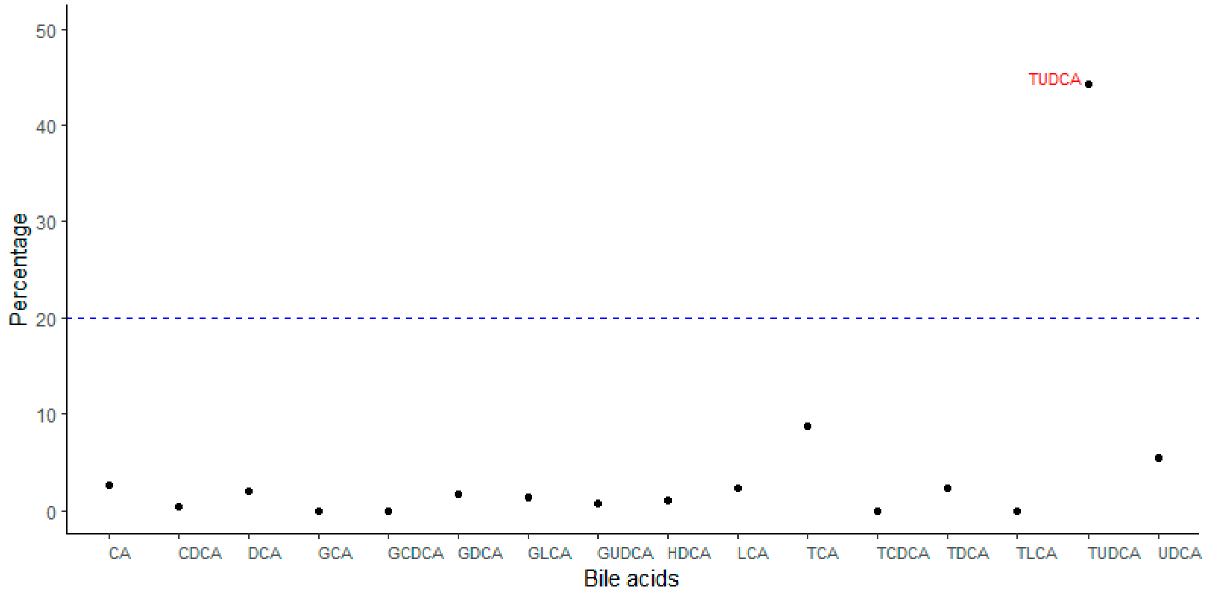

Supplementary Figure S2. Plot of the 16 bile acids according to the percentage of missingness in 309 patients with ACS. Abbreviations: CA, cholic acid; CI, confidence interval; CDCA, chenodeoxycholic acid; TCA, taurocholic acid; DCA, deoxycholic acid; GCDCA, glycochenodeoxycholic acid; GCA, glycocholic acid; GDCA, glycodeoxycholic acid; GLCA, glycolithocholic acid; GUDCA, glycoursodeoxycholic acid; HDCA, hyodeoxycholic acid; LCA, lithocholic acid; TCDCA, taurochenodeoxycholic acid; TDCA, taurodeoxycholic acid; TLCA, taurolithocholic acid; TUDCA, tauroursodeoxycholic acid; UDCA, ursodeoxycholic acid.

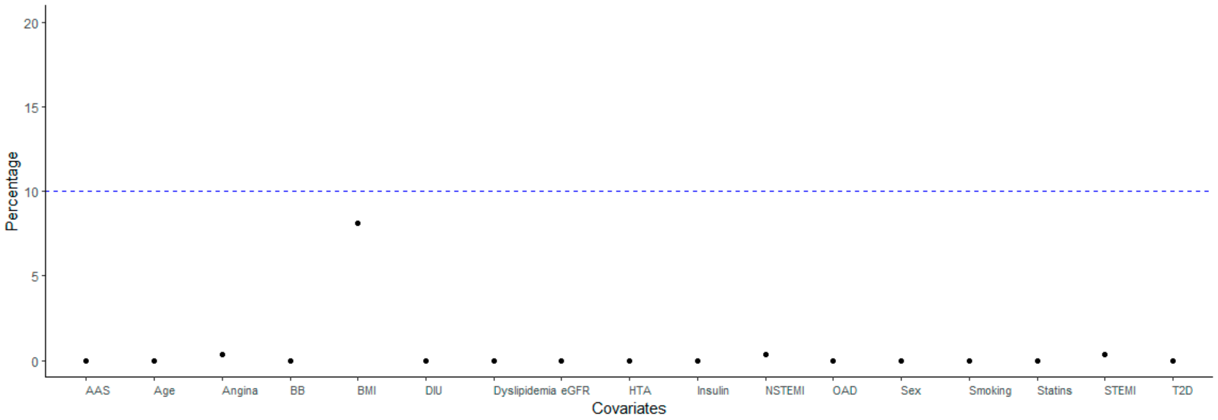

Supplementary Figure S3. Plot of the covariates according to the percentage of missingness in 309 patients with ACS. Abbreviations: AAS, aspirin; BB, beta-blockers drugs; BMI, body mass index; DIU, diuretics; eGFR, estimated glomerular filtration rate; HTA, hypertension; NSTEMI, non- ST-segment elevation myocardial infarction; OAD, oral antidiabetic drugs; STEMI, ST-segment elevation myocardial infarction; T2D, type 2 diabetes.

Supplementary Figure S4. Density plots comparing observed and imputed data.

A.

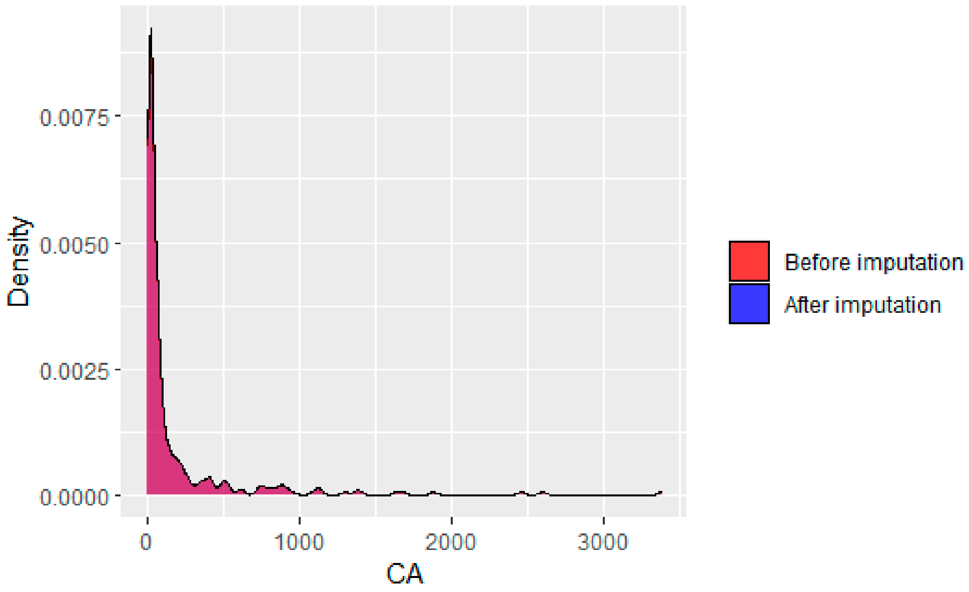

Abbreviations: CA, cholic acid.

B.

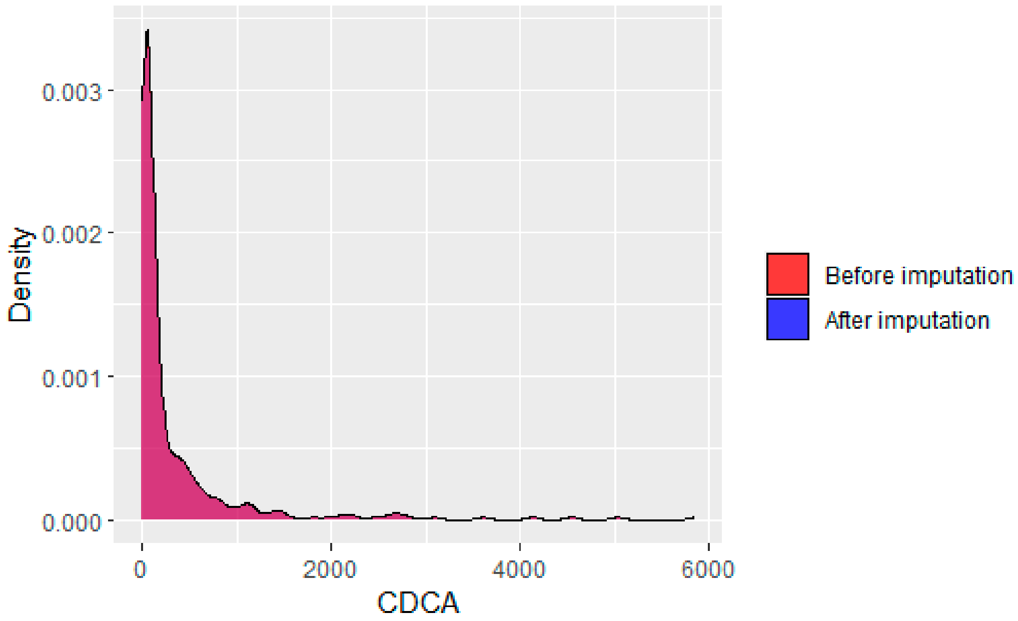

Abbreviations: CDCA, chenodeoxycholic acid.

C.

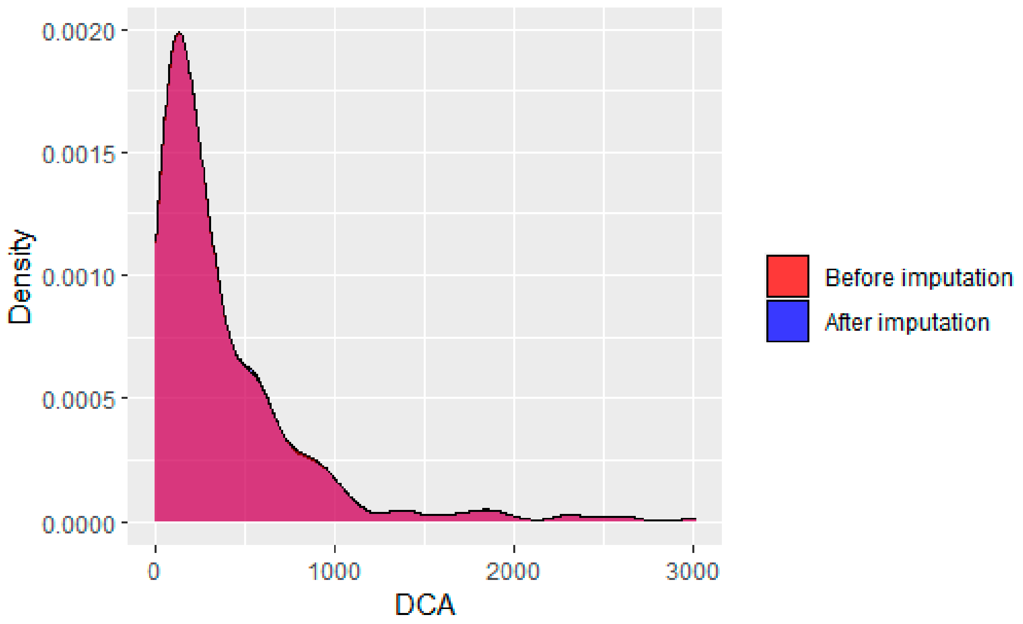

Abbreviations: DCA, deoxycholic acid

D.

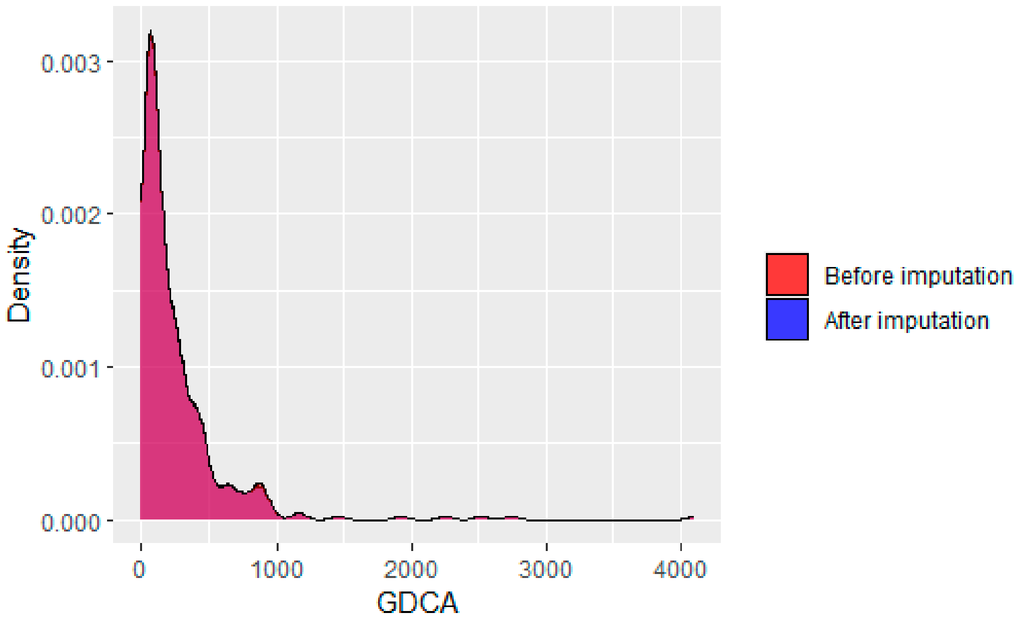

Abbreviations: GDCA, glycodeoxycholic acid.

E.

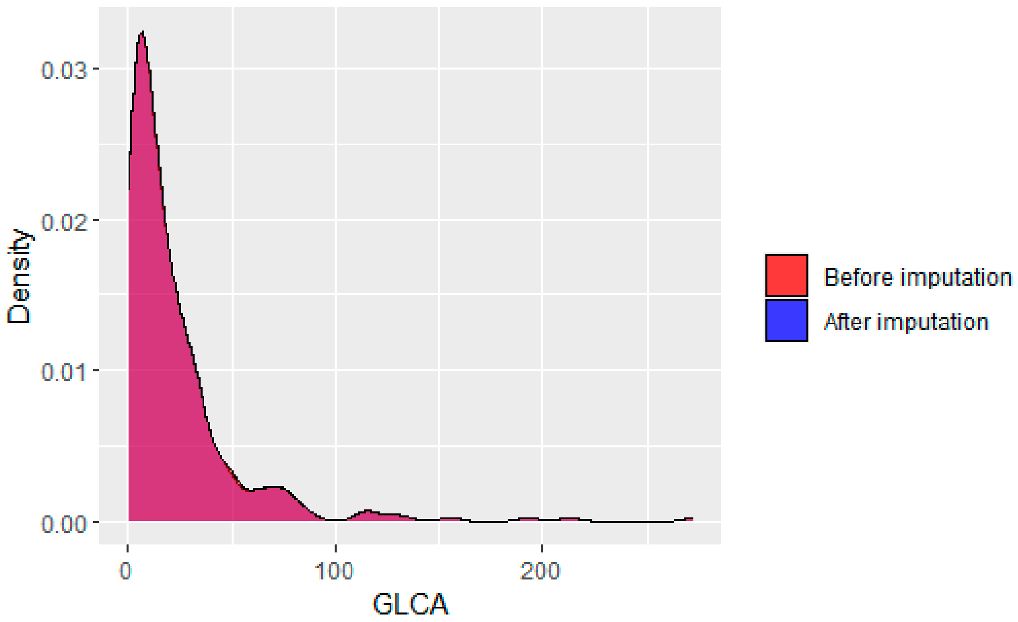

Abbreviations: GLCA, glycolithocholic acid

F.

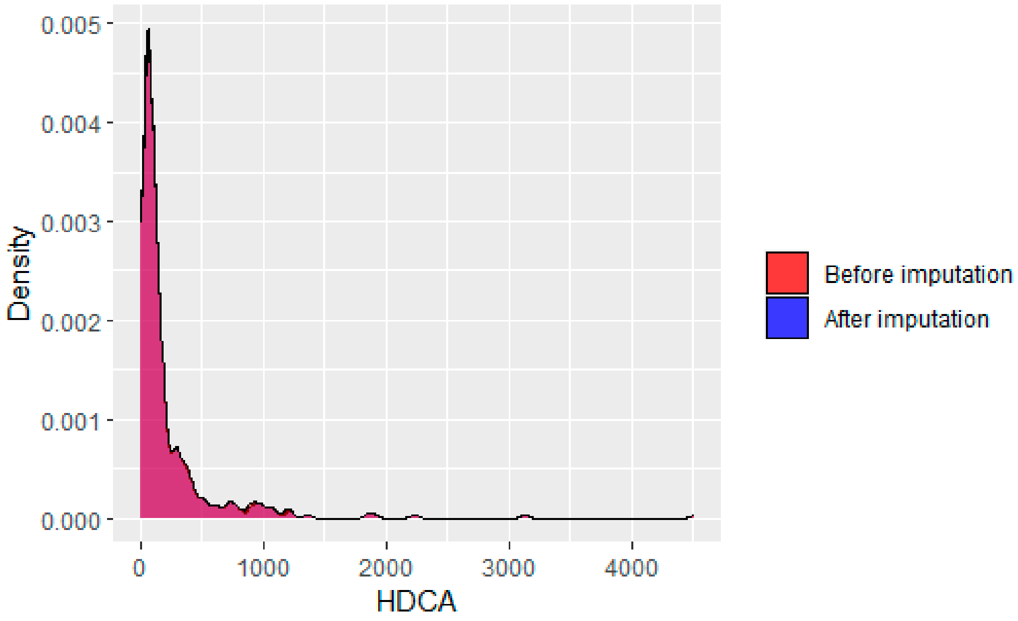

Abbreviations: HDCA, hyodeoxycholic acid.

G.

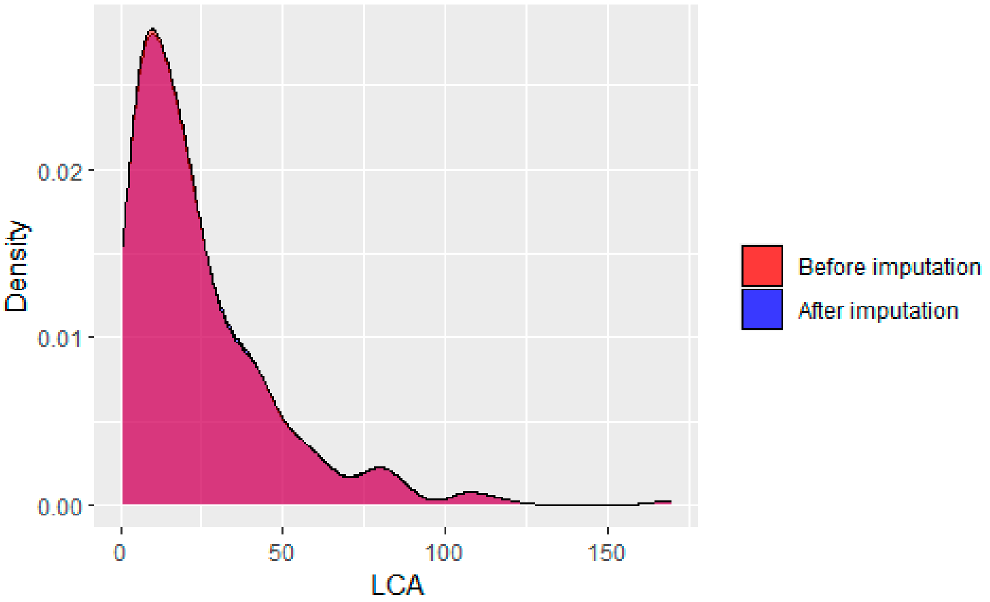

Abbreviations: LCA, lithocholic acid.

H.

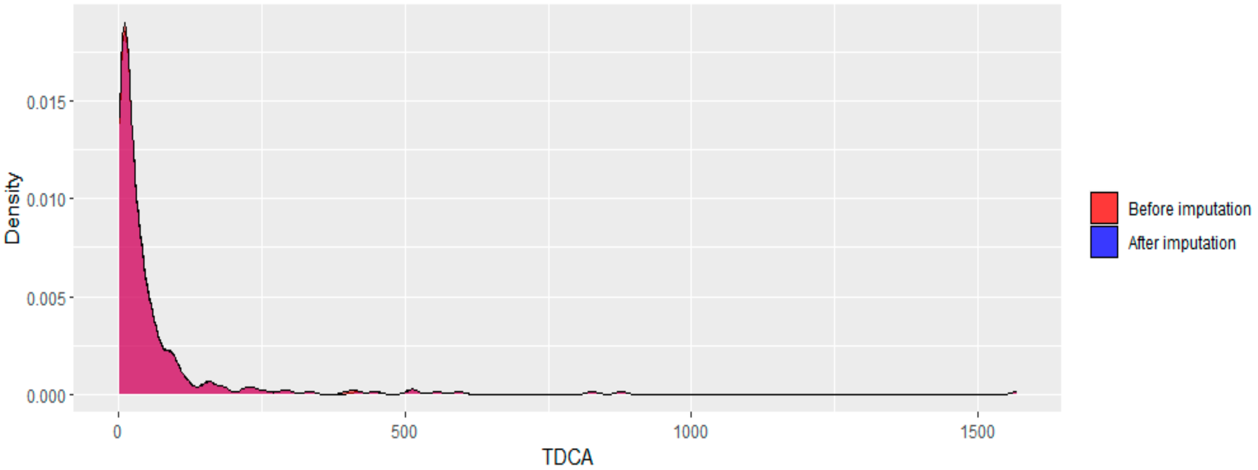

Abbreviations: TDCA, taurodeoxycholic acid.

I.

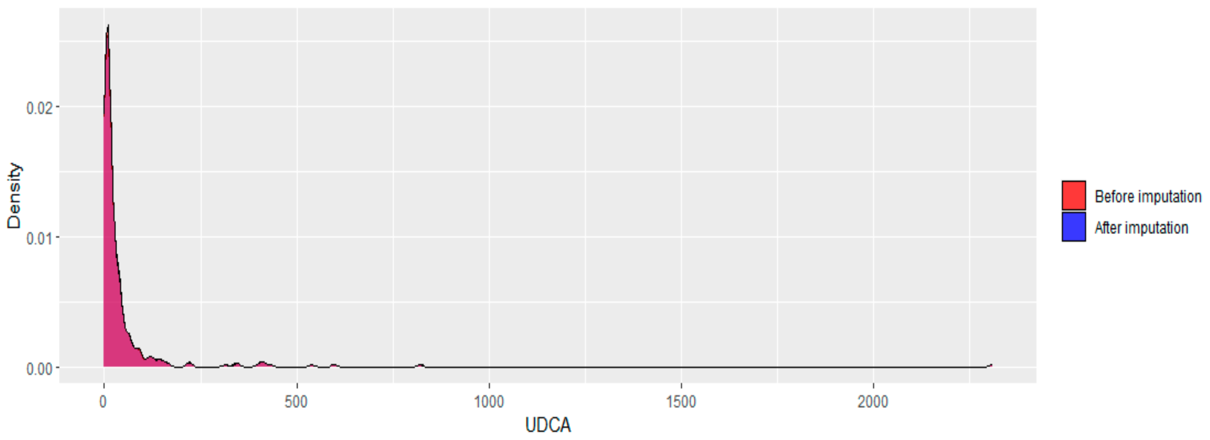

Abbreviations: UDCA, ursodeoxycholic acid.

J.

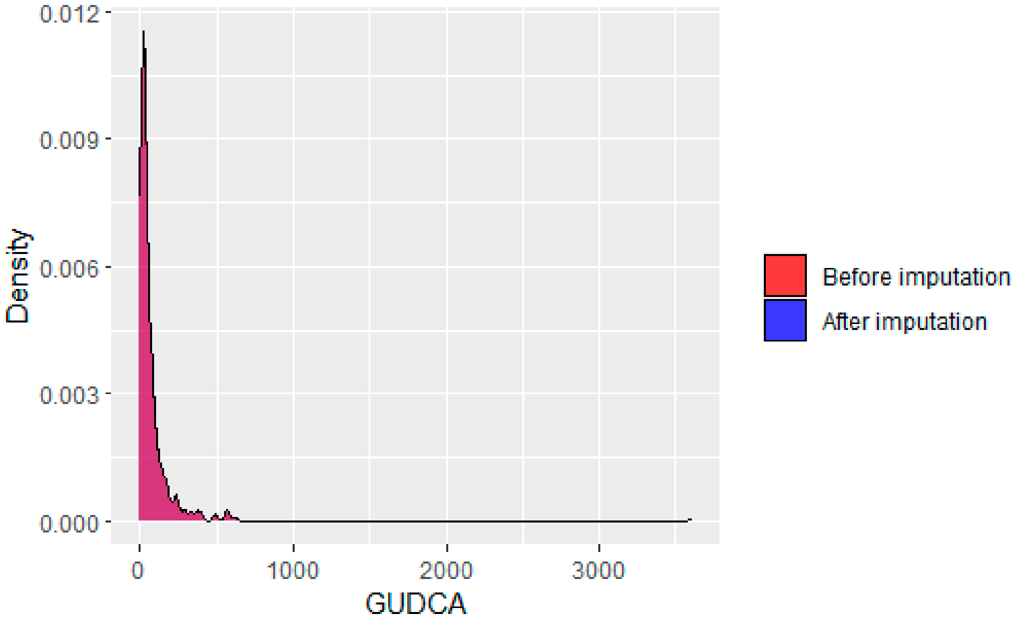

Abbreviations: GUDCA, glycoursodeoxycholic acid.

K.

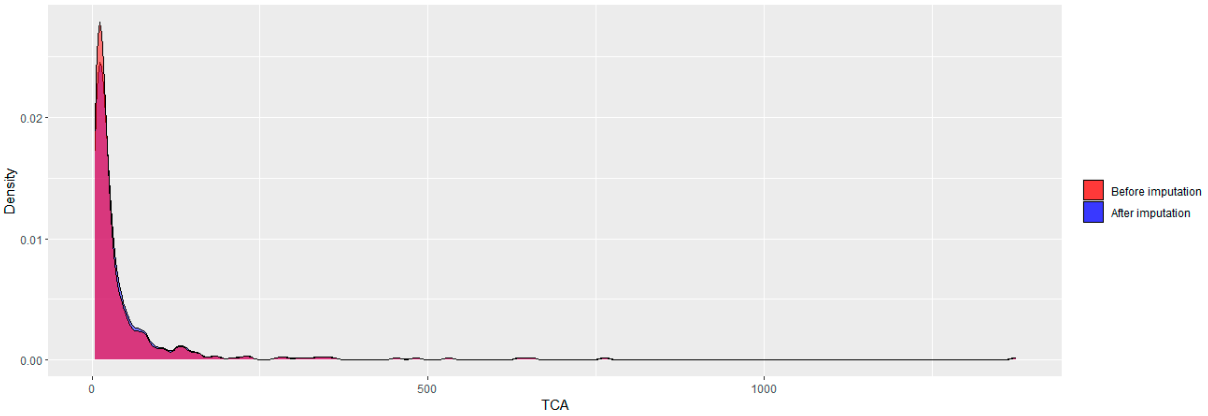

Abbreviations: TCA, taurocholic acid.

L

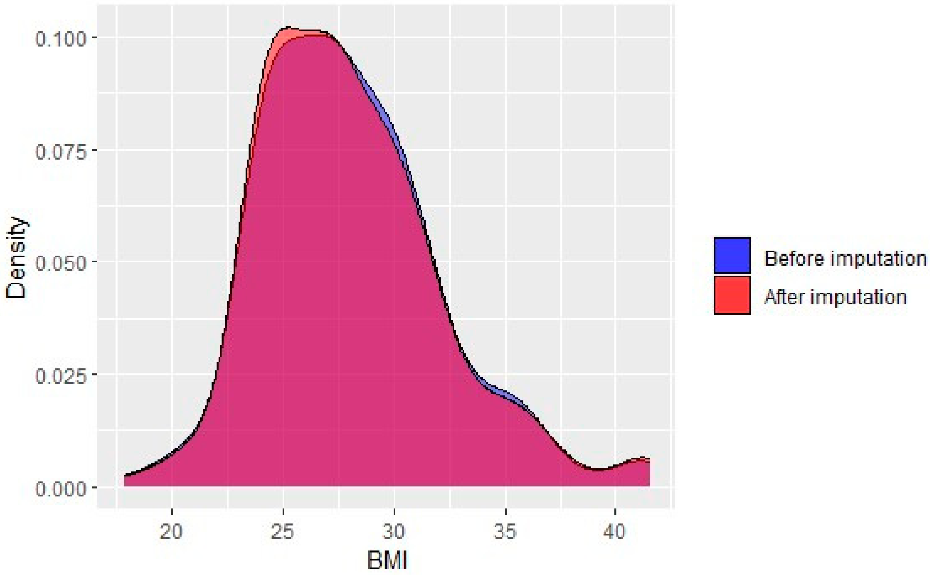

Abbreviations: BMI, body mass index.

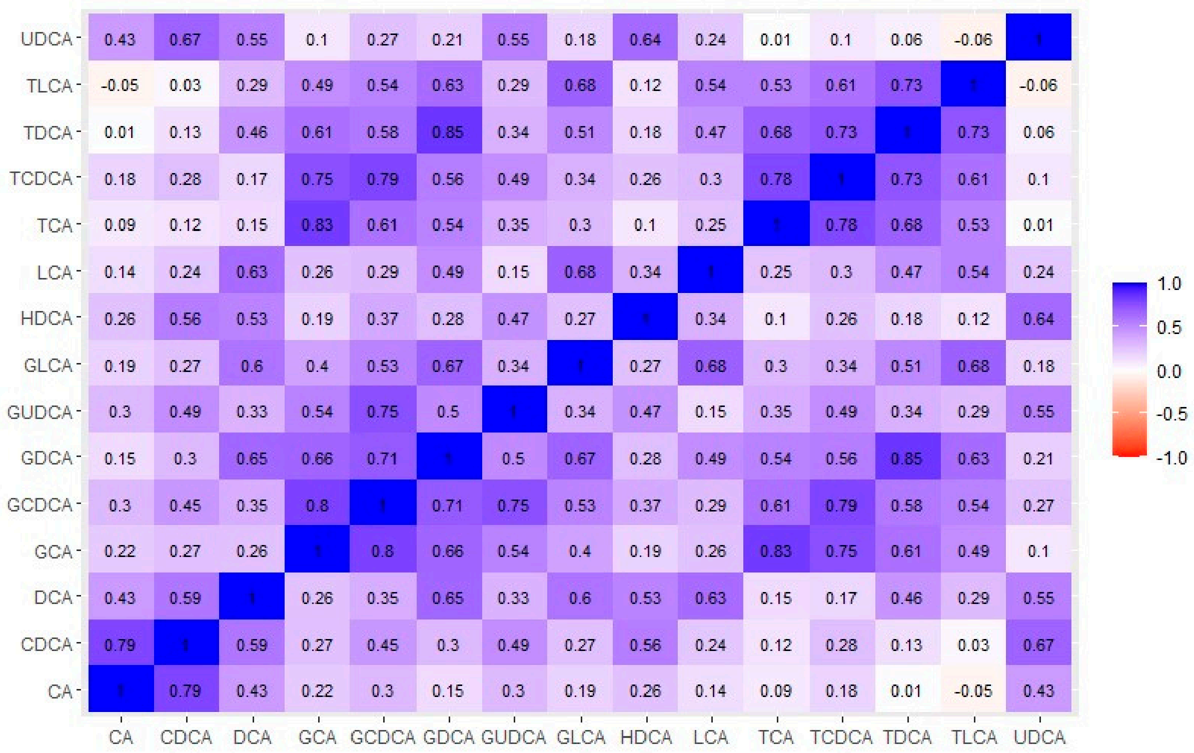

Supplementary Figure S5. Correlation matrix for the 15 bile acids considered in the analysis. Spearman's rank correlation coefficient is shown for each correlation. Abbreviations: CA, cholic acid; CI, confidence interval; CDCA, chenodeoxycholic acid; TCA, taurocholic acid; DCA, deoxycholic acid; GCDCA, glycochenodeoxycholic acid; GCA, glycocholic acid; GDCA, glycodeoxycholic acid; GLCA, glycolithocholic acid; GUDCA, glycoursodeoxycholic acid; HDCA, hyodeoxycholic acid; LCA, lithocholic acid; TCDCA, taurochenodeoxycholic acid; TDCA, taurodeoxycholic acid; TLCA, taurolithocholic acid; UDCA, ursodeoxycholic acid.

Supplementary Table S2. Baseline concentrations of the bile acids measured in ACS patients.

Bile acids, nM	**Patients (N=309)**
Primary bile acids	
CA	35.06 (14.52, 125.09)
CDCA	79.95 (28.32, 333.09)
TCA	19.54 (10.57, 46.45)
Secondary bile acids	
DCA	227.88 (109.30, 514.33)
GCDCA	400.29 (206.65, 799.74)
GCA	85.84 (45.17, 206.18)
GDCA	138.05 (56.47, 307.80)
GLCA	13.41 (5.35, 29.25)
GUDCA	38.30 (15.05, 83.01)
HDCA	93.97 (45.94, 202.23)
LCA	17.78 (8.56, 33.09)
TCDCA	71.23 (32.99, 149.76)
TDCA	22.41 (7.51, 51.12)
TLCA	3.53 (2.52, 5.37)
UDCA	14.19 (5.27 ,37.01)

Data are presented as median (interquartile range). Abbreviations: CA, cholic acid; CI, confidence interval; CDCA, chenodeoxycholic acid; TCA, taurocholic acid; DCA, deoxycholic acid; GCDCA, glycochenodeoxycholic acid; GCA, glycocholic acid; GDCA, glycodeoxycholic acid; GLCA, glycolithocholic acid; GUDCA, glycoursodeoxycholic acid; HDCA, hyodeoxycholic acid; LCA, lithocholic acid; TCDCA, taurochenodeoxycholic acid; TDCA, taurodeoxycholic acid; TLCA, taurolithocholic acid; UDCA, ursodeoxycholic acid.

Supplementary Table S3. Cox regression analysis examining the individual associations between bile acids and risk of MACE.

Bile acids	Unadjusted model			MV model		
	HR (95% CI)	*P value*	*P adj*	*HR (95% CI)*	*P value*	*P adj*
CA	0.94 (0.78, 1.13)	0.542	0.813	1.03 (0.86, 1.25)	0.677	0.820
CDCA	1.01 (0.84, 1.20)	0.881	0.940	1.06 (0.88, 1.28)	0.491	0.820
TCA	0.96 (0.81, 1.15)	0.724	0.863	0.96 (0.80, 1.14)	0.657	0.820
DCA	1.08 (0.90, 1.28)	0.382	0.808	1.06 (0.88, 1.29)	0.501	0.820
GCDCA	0.90 (0.76, 1.07)	0.264	0.808	0.91 (0.75, 1.09)	0.324	0.820
GCA	0.99 (0.83, 1.17)	0.940	0.940	0.96 (0.81, 1.15)	0.711	0.820
GDCA	0.96 (0.81, 1.14)	0.683	0.863	0.88 (0.73, 1.07)	0.227	0.820
GLCA	0.93 (0.78, 1.11)	0.485	0.808	0.91 (0.76, 1.10)	0.355	0.820
GUDCA	0.93 (0.78, 1.10)	0.403	0.808	0.96 (0.79, 1.16)	0.707	0.820
HDCA	0.92 (0.77, 1.09)	0.365	0.808	0.99(0.83, 1.19)	0.995	0.995
LCA	0.93 (0.79, 1.11)	0.459	0.808	0.91 (0.76, 1.08)	0.294	0.820
TCDCA	0.86 (0.72, 1.02)	0.093	0.808	0.89 (0.75, 1.05)	0.191	0.820
TDCA	0.90 (0.75, 1.07)	0.259	0.808	0.84 (0.70, 1.02)	0.082	0.820
TLCA	0.90 (0.76, 1.07)	0.268	0.808	0.92 (0.77, 1.10)	0.393	0.820
UDCA	0.97 (0.81, 1.15)	0.748	0.863	1.02 (0.85, 1.22)	0.786	0.840

MV adjusted for age, sex, body mass index (kg/m^2), smoking, hypertension, dyslipidemia, type 2 diabetes, unstable angina, acute ST-segment elevation myocardial infarction, non-ST-segment elevation acute myocardial infarction, statin medication, beta-blockers, oral antidiabetic medication, insulin medication, diuretics, aspirin and glomerular filtration rate. Abbreviations: CA, cholic acid; CI, confidence interval; CDCA, chenodeoxycholic acid; HR, hazard ratio; TCA, taurocholic acid; DCA, deoxycholic acid; GCDCA, glycochenodeoxycholic acid; GCA, glycocholic acid; GDCA, glycodeoxycholic acid; GLCA, glycolithocholic acid; GUDCA, glycoursodeoxycholic acid; HDCA, hyodeoxycholic acid; LCA, lithocholic acid; MACE, major adverse cardiovascular event; MV, multivariable; TCDCA, taurochenodeoxycholic acid; TDCA, taurodeoxycholic acid; TLCA, taurolithocholic acid; UDCA, ursodeoxycholic acid.

Supplementary Table S4. Bile acids ranked from the highest to the lowest elastic net positive and negative regression coefficients for MACE. Exposure contrast is per SD/z-score increase of the bile acid.

**Bile acids**	**β (95% CI)**	**Bile acids**	**β (95% CI)**
DCA	0.531 (0.506, 0.555)	CA	-0.470 (-0.489, -0.451)
CDCA	0.363 (0.342, 0.384)	TDCA	-0.412 (-0.438, -0.386)
GCA	0.359 (0.338, 0.380)	GCDCA	-0.257 (-0.284, -0.229)
GUDCA	0.040 (0.024, 0.057)	HDCA	-0.233 (-0.249, -0.218)
		UDCA	-0.123 (-0.137, -0.108)
		LCA	-0.102 (-0.118, -0.086)

Abbreviations: CA, cholic acid; CDCA, chenodeoxycholic acid; CI, confidence interval; DCA, deoxycholic acid; GCA, glycocholic acid; GCDCA, glycochenodeoxycholic acid; GUDCA, glycoursodeoxycholic acid; HDCA, hyodeoxycholic acid; LCA, lithocholic acid; MACE, major adverse cardiovascular event; TDCA, taurodeoxycholic acid; UDCA, ursodeoxycholic acid.

Supplementary Table S5. Cox regression analysis examining the individual associations between bile acids and all-cause mortality.

Bile acids	Unadjusted model			MV model		
	HR (95% CI)	*P value*	*P adj*	*HR (95% CI)*	*P value*	*P adj*
CA	1.03 (0.84, 1.27)	0.891	0.900	1.11(0.90, 1.38)	0.304	0.724
CDCA	1.07 (0.86, 1.32)	0.518	0.900	1.19 (0.95, 1.50)	0.115	0.536
TCA	0.97 (0.78, 1.20)	0.798	0.900	1.00 (0.80, 1.24)	0.997	0.997
DCA	1.22 (0.98, 1.52)	0.066	0.900	1.23 (0.96, 1.59)	0.097	0.536
GCDCA	1.07 (0.87, 1.32)	0.471	0.900	1.21 (0.94, 1.54)	0.122	0.536
GCA	1.10 (0.90, 1.35)	0.334	0.900	1.09 (0.88, 1.35)	0.395	0.740
GDCA	1.12 (0.91, 1.38)	0.268	0.900	1.07 (0.84, 1.37)	0.556	0.781
GLCA	1.11 (0.90, 1.37)	0.309	0.900	1.11 (0.89, 1.39)	0.338	0.724
GUDCA	1.07 (0.87, 1.30)	0.501	0.900	1.19 (0.94, 1.51)	0.143	0.536
HDCA	0.94 (0.76, 1.16)	0.578	0.900	1.06 (0.85, 1.31)	0.581	0.781
LCA	1.04 (0.84, 1.28)	0.711	0.900	1.05 (0.84, 1.31)	0.658	0.781
TCDCA	0.92 (0.74, 1.13)	0.445	0.900	1.03 (0.83, 1.29)	0.734	0.786
TDCA	0.98 (0.79, 1.21)	0.879	0.900	0.95 (0.75, 1.19)	0.670	0.781
TLCA	0.98 (0.79, 1.22)	0.900	0.900	1.04 (0.83, 1.30)	0.677	0.781
UDCA	1.03 (0.84, 1.27)	0.753	0.900	1.15 (0.91, 1.45)	0.221	0.663

MV adjusted for age, sex, body mass index (kg/m^2), smoking, hypertension, dyslipidemia, type 2 diabetes, unstable angina, acute ST-segment elevation myocardial infarction, non-ST-segment elevation acute myocardial infarction, statin medication, beta-blockers, oral antidiabetic medication, insulin medication, diuretics, aspirin and glomerular filtration rate. Abbreviations: CA, cholic acid; CI, confidence interval; CDCA, chenodeoxycholic acid; HR, hazard ratio; TCA, taurocholic acid; DCA, deoxycholic acid; GCDCA, glycochenodeoxycholic acid; GCA, glycocholic acid; GDCA, glycodeoxycholic acid; GLCA, glycolithocholic acid; GUDCA, glycoursodeoxycholic acid; HDCA, hyodeoxycholic acid; LCA, lithocholic acid; MV, multivariable; TCDCA, taurochenodeoxycholic acid; TDCA, taurodeoxycholic acid; TLCA, taurolithocholic acid; UDCA, ursodeoxycholic acid.

Supplementary Table S6. Bile acids ranked from the highest to the lowest elastic net positive and negative regression coefficients for all-cause mortality. Exposure contrast is per SD/z-score increase of the bile acid.

**Bile acids**	**β (95% CI)**	**Bile acids**	**β (95% CI)**
DCA	0.432 (0.412, 0.452)	CA	-0.315 (-0.331, -0.299)
GCA	0.335 (0.314, 0.355)	HDCA	-0.282 (-0.300, -0.265)

Abbreviations: CA, cholic acid; CI, confidence interval; DCA, deoxycholic acid; GCA, glycocholic acid; HDCA, hyodeoxycholic acid.
